# Confronting Subtle Workplace Mistreatment: The Importance of Leaders as Allies

**DOI:** 10.3389/fpsyg.2017.01051

**Published:** 2017-06-23

**Authors:** Kimberly T. Schneider, Eric D. Wesselmann, Eros R. DeSouza

**Affiliations:** Department of Psychology, Illinois State UniversityNormal, IL, United States

**Keywords:** workplace mistreatment, derogatory humor, incivility, workplace harassment, allies, organizational climate, ally training

Workplace discrimination, harassment, exclusion, and incivility incidents are often subtle in nature but can nonetheless have deleterious impacts on targets (Zurbrügg and Miner, 2016). Additionally, there are often few (if any) clearly defined social norms regarding these types of behaviors in organizations (DeSouza, 2011). Interventions targeting the subtle aspects of workplace mistreatment may not only be a fruitful avenue for reducing or eliminating the development of these types of mistreatment, but may also prevent more overt forms of negative workplace interactions (Jones et al., 2017). Because organizational leaders are sometimes bystanders to mistreatment, we propose that organizations should develop leaders into *allies* who are trained to intervene as well as to develop and clarify workplace norms prohibiting subtle forms of targeted workplace mistreatment. We generalize specifically from research on discrimination against LGBT employees, because they have received little legal protection until recently, as well as on best practices for providing support to these employees and propose that initiatives used recently by both unions and universities can provide insights into the effectiveness of allies in creating organizational climate change.

An ally is typically defined as someone who supports the reduction of homophobia and actively provides support and equality for LGBT individuals. More generally, we extend this term to involve someone who is identifiable by their commitment to challenging interpersonal and institutional forms of workplace mistreatment against a member of any minority group. When allies are bystanders to subtle mistreatment, they can use this commitment to challenge perpetrators and illuminate biases.

## The influence of allies in changing climates

Leaders can become impactful allies when they prioritize creating an inclusive organizational climate. When minority group members feel unsupported or excluded, they often feel directly marginalized due to their identities. Research suggests that LGBT employees suffer psychologically when they conceal their sexual orientations at work for fear of mistreatment (Ruggs et al., 2015). Additionally, some LGBT employees and students who conceal their sexual orientations engage in emotional labor, expending considerable cognitive energy to self-regulate behaviors and manage impressions. Gay and gender non-conforming university students have described feeling marginalized through the unknowing comments of peers; these students reported monitoring their emotions to regulate their behavior (Toynton, 2007) and this type of constant self-monitoring can negatively impact performance.

Depending on group climate, day-to-day workplace interactions may be rife with incivility, micro-aggression, and subtle discrimination (including homophobic jokes or keeping marginalized employees “out of the loop”) that signal to LGBT employees to stay in the closet. Additionally, a lack of inclusive organizational policies and the presence of heterosexist norms and language (e.g., differential references to “spouses” as opposed to “friends” or “roommates” based on employees' sexual orientations) can serve as subtle cues illustrating an unsupportive climate even if a particular employee is not directly targeted (DeSouza et al., 2017). Employees may withdraw from social or work-related interactions with colleagues to avoid potential subtle discrimination (DeSouza et al., 2017) or due to a fear of being accidentally “outed” by LGBT acquaintances, friends, or loved ones (Ragins et al., 2007). Such avoidance can negatively impact networking or other career opportunities.

However, if organizational leaders step up as allies, they can function similarly to university faculty and staff who have created safe spaces to offer support to minority students. Organizational leaders can set a climate that emphasizes the importance of inclusion. Sexual harassment researchers have established links between an organization's tolerance of harassment and the prevalence of harassment; supervisors who signal tolerance of harassment vis-à-vis skepticism of targets' reports, along with weak or nonexistent sanctions for perpetrators, are leaders in groups where harassment prevalence is higher (Hulin et al., 1996; DeSouza, 2011). Thus, leaders' attitudes toward harassment and their enforcement of organizational policies and procedures generate the organization's climate. Similar processes likely occur for other types of subtle workplace mistreatment.

## Allies as bystanders: instances of derogatory humor

Supervisors commonly serve as organizational educators; thus, employees may be open to feedback from a supervisor who confronts subtle mistreatment rather than ignoring such behavior. When supervisors are bystanders to questionable interactions they should immediately “call out” such behavior in order to change attitudes and clearly signal to other employees that it is not tolerated. Even brief comments (e.g., “not cool” or “ouch”) provide informative feedback that can influence a perpetrator to rethink displays of subtle bias.

We want to emphasize the risk of ignoring mistreatment. At a minimum, silent bystanders can intentionally or unintentionally add to targets' feelings of ostracism (e.g., Chernyak and Zayas, 2010) and they can contribute to a climate of intolerance. Consider disparaging humor that includes sexist, racist, or homophobic jokes; this humor can be deceptive because bystanders often do not perceive the joke-teller as intentionally prejudiced and they might respond by either laughing or refraining from challenging the joke-teller. These responses can later justify more mistreatment toward the target group because they indicate to others that discrimination is socially acceptable (Mallett et al., 2016). Disparaging humor may appear to not target particular employees, yet it still trivializes and stigmatizes an entire group based on its identity and can foster a prejudiced normative climate, especially if the joke-teller has aggressive motives and uses humor to assert dominance (Hodson and MacInnis, 2016). If the joking is homophobic in nature, such jokes may be particularly influential given that there is often ambiguity surrounding norms for the (un)acceptability of homophobia due to inconsistent workplace anti-discrimination laws (DeSouza et al., 2017). Leaders who confront displays of subtle bias, such as disparaging humor, can help clarify ambiguous situations, putting them in powerful advocacy positions.

## Resources for ally support

Organizational leaders and HR professionals can use labor union advocacy initiatives to develop approaches for training allies. Specific to LGBT advocacy, *Pride at Work* is a nonprofit constituency group within the AFL-CIO that provides support to LGBT union members and allies. Along with opposing both workplace and union discrimination against employees on the basis of sex, gender identity, sexual orientation, race, national origin, age, disability, religion, or political views, *Pride at Work* also engages in union activism for the LGBT community. Their website (www.prideatwork.org) provides resources related to workplace discrimination laws and applications to union issues such as suggestions for LGBT-inclusive contract language. Another example of effective union advocacy is a recent collaboration between the International Brotherhood of Electrical Workers, the Communications Workers Association, and Verizon that trained male allies to combat domestic violence against women by focusing on changes in workplace climate (Wagner et al., 2012).

Organizational leaders can also draw from university-based advocacy efforts. As noted earlier, at many universities, “*Safe Zones”* are designated areas on campus where allies publicly state their support for LGBT students. In recognizing locations and individuals who are available for assistance, students are afforded tangible social support linked directly to their institutions. In addition to Safe Zone training, other institution-specific committees, panels, and training programs often exist. For example, at our university, an *Inclusive Community Response Team* (ICRT) acts as a task force to foster inclusiveness by investigating complaints related to hate or bias reported by university community members. The ICRT includes representatives from campus police, the Dean of Students office, the university housing office, and student counseling services; it uses expertise from various domains of campus life to determine if bias-based violations of the Student Code of Conduct or criminal acts have occurred, as well as broader concerns about campus climate and accessibility for all students. Other campus initiatives, such as the *Triangle Association*, focus on improving the retention of LGBT faculty and staff. If such campus initiatives are highly visible and demonstrate their effectiveness in creating change, individuals predisposed to engage in subtle discrimination likely receive cues that such behavior is unacceptable. Such initiatives may also increase a general feeling of inclusion for members of marginalized groups.

Organizational leaders may also create *affinity groups* (employees linked by a common interest) in order to facilitate networks of supportive individuals with diverse identities and backgrounds. These groups provide opportunities for intergroup interaction that highlight similarities among members on the basis of their shared interest, a solution that is known to improve empathy. However, we argue that simple social contact, although a useful strategy, works best when individuals have opportunities to demonstrate their expertise and move beyond simple interactions. For instance, unfamiliarity is not likely to be the main driver of gender-based discrimination; in some workplaces, the same could be said for race-based, sexual orientation, or religious-based subtle discrimination. Additionally, even in affinity groups, it is difficult to eliminate pre-existing group status and power differences that often limit what is gained from intergroup contact. Workplace strategies focusing on intergroup interactions should be paired with other approaches that reduce status and power differences and allow task- and skill-based demonstrations of diverse group members' expertise.

## Potential barriers and a call for further research

Organizational leaders can increase both the inclusivity of their workplaces and the wellbeing of their employees. However, allies' effectiveness may be limited based on their perceived motives. A supervisor may be perceived as completing ally training or confronting bias for self-serving motives, rather than out of concern for equality. Worse, allies' behavior may be perceived as paternalistic or they may be resented as “outsiders” (Duhigg et al., 2010). Allies can refute such concerns by describing their goals related to creating a supportive tone that provides equal opportunities for optimal well-being. This approach may also make resistant employees (or potential perpetrators of mistreatment) more accepting of allies, given that employee well-being is generally highly valued.

Organizations should assess potential barriers before implementing ally programs, such as the time and resources it may take to change organizational culture. Leaders should be prepared to address backlash if ally programs are perceived negatively by majority group members or if affinity groups are perceived as exclusionary. Ally training should also prepare trainees for potential backlash (Ashburn-Nardo et al., 2008; Good et al., 2012) and negative emotions reported by some allies such as guilt due to a sense of not doing enough to help, or disappointment in others who do not support their efforts (Asta and Vacha-Haase, 2013).

Little research has assessed ally training, but general diversity training assessments indicate medium-to-large effects on cognitive-based and skill-based outcomes, and small-to-medium effects on affective outcomes (Kalinoski et al., 2013). There may be ceiling effects for ally training programs, given that many employees with supportive attitudes toward diversity will self-select into training programs. Counselors who had recently completed discussion-based LGBT ally training indicated in post-training interviews that self-awareness of their behavioral and advocacy efforts had been improved, but there was no evidence of training-based attitudinal change, perhaps due to pre-existing positive attitudes (Rivers and Swank, 2017). Such results are consistent with meta-analytic evidence of larger effects of diversity training on trainee self-efficacy than on attitudes (Kalinoski et al., 2013).

There is a clear need for additional empirical studies within organizations that directly examine the processes by which allies can change intolerant climates. Effective ally strategies may depend on the specific group targeted for mistreatment, along with the perpetrator's and ally's demographic characteristics, status, or power (Ashburn-Nardo et al., 2008; Drury and Kaiser, 2014). For instance, male leaders who confronted perpetrators were viewed more favorably when they used public strategies as opposed to private strategies, whereas female leaders who were indirect and private in confrontations were perceived more favorably (Gervais and Hillard, 2014). This evidence would be useful in adapting ally training to certain contexts (e.g., male-dominated organizations). The resources exist to better position leaders to be effective allies; now we need to learn more about particular strategies that best create climate change.

## Author contributions

All authors listed have made substantial, direct and intellectual contribution to the work, and approved it for publication.

### Conflict of interest statement

The authors declare that the research was conducted in the absence of any commercial or financial relationships that could be construed as a potential conflict of interest.
